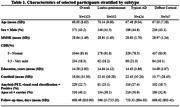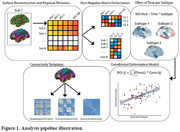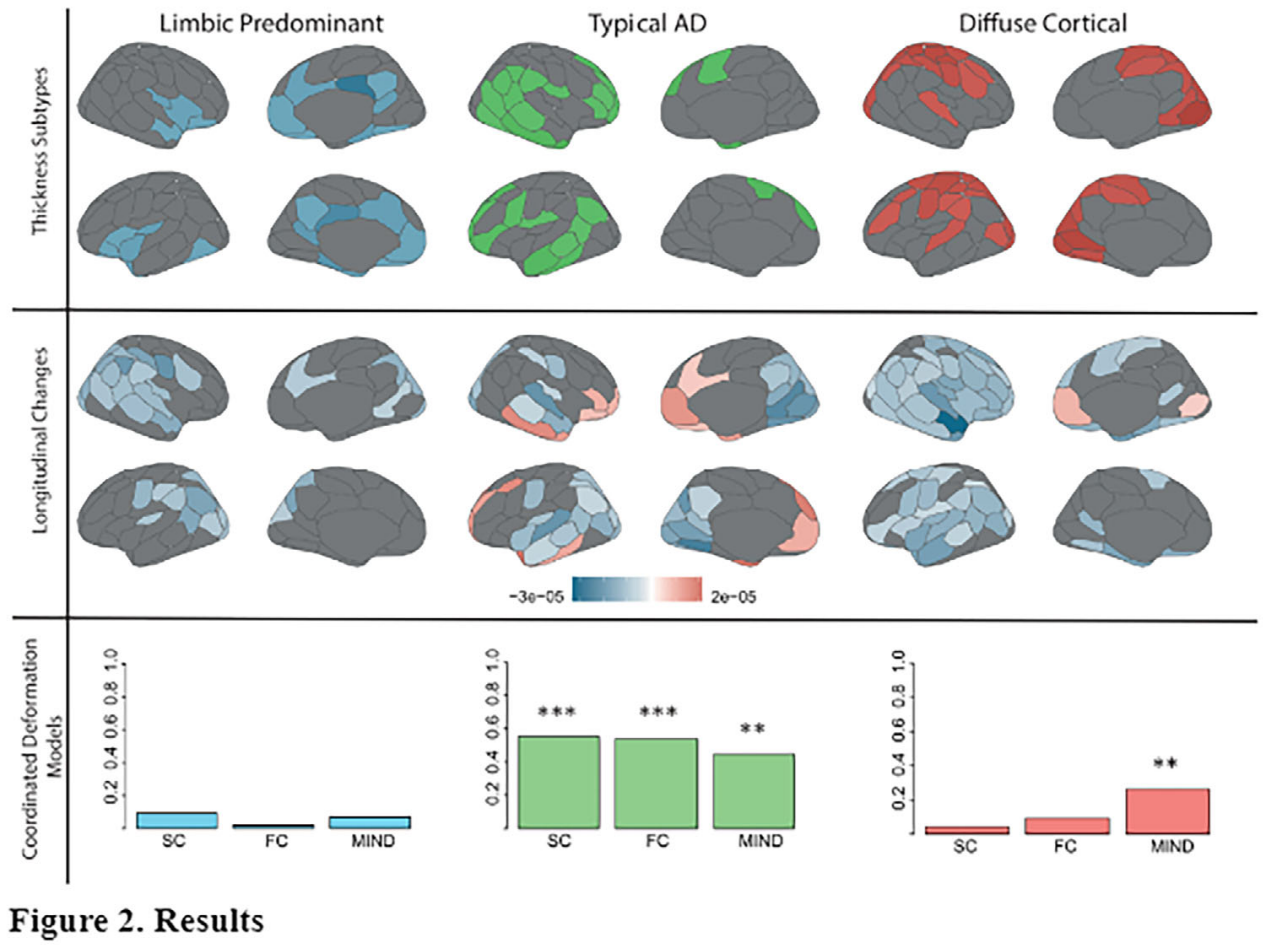# Differential network‐based propagation of cortical thinning in atrophy subtypes in non‐demented individuals

**DOI:** 10.1002/alz.093975

**Published:** 2025-01-09

**Authors:** Luigi Lorenzini, Mario Tranfa, Leonard Pieperhoff, Mara ten Kate, Giuseppe Pontillo, Isadora Lopes Alves, Mahnaz Shekari, Daniele Altomare, Anouk den Braber, Craig W Ritchie, Mercè Boada, Marta Marquié, Rik Vandenberghe, Emma S. Luckett, Bernard J Hanseeuw, Pieter Jelle Visser, Michael Schöll, Giovanni B. Frisoni, Cindy Birck, Anja Mett, Andrew W. Stephens, Rossella Gismondi, Christopher Buckley, Gill Farrar, Frank Jessen, Juan Domingo Gispert, Henk‐Jan Mutsaerts, David Vállez García, Alle Meije Wink, Lyduine E. Collij, Frederik Barkhof

**Affiliations:** ^1^ Department of Radiology and Nuclear Medicine, Amsterdam Neuroscience, Vrije Universiteit Amsterdam, Amsterdam UMC, Amsterdam Netherlands; ^2^ University of Naples Federico II, Naples Italy; ^3^ Amsterdam UMC, Amsterdam Netherlands; ^4^ Department of Neurology, Alzheimer Center Amsterdam, Amsterdam Neuroscience, Vrije Universiteit Amsterdam, Amsterdam Netherlands; ^5^ Department of Electrical Engineering and Information Technology, University of Naples “Federico II”, Naples Italy; ^6^ Brain Research Center, Amsterdam Netherlands; ^7^ Barcelona?eta Brain Research Center (BBRC), Pasqual Maragall Foundation, Barcelona Spain; ^8^ University of Geneva, Geneva Switzerland; ^9^ Centre for Clinical Brain Sciences, The University of Edinburgh, Edinburgh United Kingdom; ^10^ Ace Alzheimer Center, Barcelona Spain; ^11^ Ace Alzheimer Center Barcelona, Barcelona Spain; ^12^ University Hospitals Leuven, Leuven Belgium; ^13^ Laboratory for Cognitive Neurology, Leuven Brain Institute, KU Leuven, Leuven Belgium; ^14^ Institute of Neuroscience ‐ UCLouvain, Brussels Belgium; ^15^ Alzheimer Center and Department of Neurology, Amsterdam Neuroscience Campus, VU University Medical Center, Amsterdam Netherlands; ^16^ University of Gothenburg, Gothenburg Sweden; ^17^ Alzheimer Europe, Luxembourg Luxembourg; ^18^ GE Healthcare, Amersham United Kingdom; ^19^ Life Molecular Imaging GmbH, Berlin Germany; ^20^ GE HealthCare, Amersham United Kingdom; ^21^ University of Cologne, Faculty of Medicine and University Hospital Cologne, Cologne Germany; ^22^ Lund University, Lund Sweden

## Abstract

**Background:**

Different patterns of atrophy exist in the dementia stage of AD. However, little is known about the heterogeneity of atrophy patterns and the mechanisms that drive subsequent propagation of the disease in the preclinical stages.

**Method:**

From the AMYPAD‐PNHS cohort, we included a total of 1323 non‐demented individuals, including 1094 amyloid‐negative, and 229 amyloid‐positive participants (Table 1). Gray matter thickness in 100 regions of interest was measured using FreeSurfer. Global cortical amyloid burden was derived from amyloid PET scans in Centiloids (CL). Individuals were assigned to atrophy subtypes based on cortical thickness measures using Non‐Negative Matrix Factorization (Figure 1). Multinomial regression models were used to study differences between subtypes in age, sex, global CL and APOE E4 carriership. Linear regression models were used to investigate the effect of time on atrophy within each subtype. Coordinated deformation models (Figure 1, Shafiei et al. 2023) were used to estimate network‐based thickness changes in each region, by multiplying the effect of time in connected regions by the strengths of their connections. Connectivity strength was based on templates of functional, structural and morphometric similarity networks, and allowed us to evaluate differential mechanisms. Significance was tested using a permutation approach.

**Results:**

Three significant subtypes of regional GM atrophy were identified:‘limbic predominant’, ‘typical AD’, and ‘diffuse cortical’ (Figure 2). Individuals assigned to the limbic predominant subtype were significantly older, while the typical AD subtype had more APOE E4 carriers compared to the others (all p<0.001). Both limbic predominant and typical AD subtypes had higher CL compared to the diffuse cortical atrophy subtype (all p<0.001). Significant longitudinal thickness changes per subtype are shown in Figure 2. In the typical AD subtype, all network architectures constrained longitudinal GM changes (p<0.001), while in the diffuse atrophy subtype, propagation was mostly determined by morphometric similarity (p<0.005). No significant prediction model was found for the limbic predominant subtype.

**Conclusion:**

We show that atrophy subtypes can already be detected in non‐demented individuals. Progression of atrophy within subtypes is determined by network‐based mechanisms.